# Fluopsin C for Treating Multidrug-Resistant Infections: *In vitro* Activity Against Clinically Important Strains and *in vivo* Efficacy Against Carbapenemase-Producing *Klebsiella pneumoniae*

**DOI:** 10.3389/fmicb.2019.02431

**Published:** 2019-10-25

**Authors:** Miguel Octavio Pérez Navarro, Ane Stefano Simionato, Juan Carlos Bedoya Pérez, André Riedi Barazetti, Janaina Emiliano, Erika Tyemi Goya Niekawa, Matheus Felipe de Lima Andreata, Fluvio Modolon, Mickely Liuti Dealis, Eduardo José de Almeida Araújo, Thalita Massi Carlos, Odair José Scarpelim, Denise Brentan da Silva, Andreas Lazaros Chryssafidis, Per Bruheim, Galdino Andrade

**Affiliations:** ^1^Microbial Ecology Laboratory, Department of Microbiology, State University of Londrina, Londrina, Brazil; ^2^Institución Universitaria Colegio Mayor de Antioquia, Medellín, Colombia; ^3^Department of Histology, State University of Londrina, Londrina, Brazil; ^4^Biological and Health Sciences Centre, Federal University of Mato Grosso do Sul, Campo Grande, Brazil; ^5^Veterinary Toxicology Laboratory, Department of Preventive Veterinary Medicine, State University of Londrina, Londrina, Brazil; ^6^Department of Biotechnology and Food Science, NTNU – Norwegian University of Science and Technology, Trondheim, Norway

**Keywords:** antibiotic, murine sepsis model, resistant mutant, electronic microscopy, histopathology, metalloantibiotic

## Abstract

The increasing emergence of multidrug-resistant (MDR) organisms in hospital infections is causing a global public health crisis. The development of drugs with effective antibiotic action against such agents is of the highest priority. In the present study, the action of Fluopsin C against MDR clinical isolates was evaluated under *in vitro* and *in vivo* conditions. Fluopsin C was produced in cell suspension culture of *Pseudomonas aeruginosa* LV strain, purified by liquid adsorption chromatography and identified by mass spectrometric analysis. Bioactivity, bacterial resistance development risk against clinically important pathogenic strains and toxicity in mammalian cell were initially determined by *in vitro* models. *In vivo* toxicity was evaluated in *Tenebrio molitor* larvae and mice. The therapeutic efficacy of intravenous Fluopsin C administration was evaluated in a murine model of *Klebsiella pneumoniae* (KPC) acute sepsis, using six different treatments. The *in vitro* results indicated MIC and MBC below 2 μg/mL and low bacterial resistance development frequency. Electron microscopy showed that Fluopsin C may have altered the exopolysaccharide matrix and caused disruption of the cell wall of MDR bacteria. Best therapeutic results were achieved in mice treated with a single dose of 2 mg/kg and in mice treated with two doses of 1 mg/kg, 8 h apart. Furthermore, acute and chronic histopathological studies demonstrated absent nephrotoxicity and moderate hepatotoxicity. The results demonstrated the efficacy of Fluopsin C against MDR organisms in *in vitro* and *in vivo* models, and hence it can be a novel therapeutic agent for the control of severe MDR infections.

## Introduction

The worldwide, intense and frequent use of antimicrobials is a selective pressure for resistant bacteria. The fast emergence of resistant microorganisms inside hospitals is causing a global health crisis, impairing the efficacy of antibiotics and jeopardizing the success of medical treatments. Tackling antimicrobial resistance (AMR), which includes multidrug resistance (MDR), is the main goal in order to increase treatment success and lower the number of deaths of severe hospital-acquired infections. The discovery and development of novel antibiotic molecules and compounds, with high activity against untreatable MDR bacteria, is necessary (Ventola, 2015). Vancomycin-resistant *Enterococcus* (VRE), methicillin-resistant *Staphylococcus aureus* (MRSA), and Carbapenem-resistant Enterobacteria (CRE), including Carbapenemase-producing *Klebsiella pneumoniae* (KPC), are among the greatest risks for health services due to their increasing rates of antibiotic resistance (Boucher et al., 2009; Arias and Murray, 2012).

The development of molecules complexed with metals as potential medicinal agents has been rising the last decade (Cardozo et al., 2013; de Oliveira et al., 2016; Kerbauy et al., 2016; Munhoz et al., 2017; Stringer et al., 2017). Previous studies demonstrated that specific fractions obtained from the culture of *Pseudomonas aeruginosa* LV strain (F3, F3D, and F4A) present very strong antimicrobial activity against many different pathogens (de Oliveira et al., 2011, 2016; Cardozo et al., 2013; Murate et al., 2015; Kerbauy et al., 2016; Munhoz et al., 2017; Simionato et al., 2017). Indeed, our research group demonstrated the strong antibiotic activity of these semi-purified fractions against planktonic cells and biofilm formation of MDR isolates (Kerbauy et al., 2016). The gene expression analysis of *P. aeruginosa* LV strain showed that, when it is cultured in the presence of copper chloride, the bioremediation of intracellular excess copper forms a compound with high antimicrobial activity (Gionco et al., 2017). This bioactive compound was identified as a metalloantibiotic (organocopper compound), which is a great candidate for the development of new antibiotics to control infections caused by MRSA, CRE, VRE, and KPC-producing strains (Cardozo et al., 2013; Kerbauy et al., 2016).

In the present study, this metalloantibiotic was identified as Fluopsin C (YC 73), a compound produced and isolated from *Pseudomonas* spp. and *Streptomyces* sp., with high antibacterial, antifungal and antitumor activity (Itoh et al., 1970; Otsuka et al., 1972; Ma et al., 2013). However, there is a lack of studies on microbial resistance development, ultrastructural effect in the target pathogens and *in vivo* efficacy of Fluopsin C, which may confirm the possible application of this compound as an alternative for the treatment of severe human infections.

New studies on the evaluation the bioactivity, resistance-development risk, toxicity and therapeutic efficacy of Fluopsin C are required to determine the suitability of its therapeutic application. Therefore, the objective of the present study was to verify the toxicity and the effects against MDR bacteria of Fluopsin C using *in vitro* and *in vivo* experiments. Mammalian blood and cells were used to determine the hemolytic and cytotoxic effects of the compound. *Tenebrio molitor* larvae were used to evaluate Fluopsin C lethal concentration. The therapeutic efficacy against carbapenemase-producing *K. pneumonia* (KPC-KP), as well as the hepatotoxic and nephrotoxic effects, were analyzed in a murine sepsis model.

## Materials and Methods

### Microorganisms

The Fluopsin C was produced by *P. aeruginosa* LV strain (GenBank: QBLE00000000.1). These microorganisms were isolated from a citrus canker lesion on an orange (*Citrus sinensis* cv.Valence) at Astorga, Paraná, Brazil (Gionco et al., 2017). The pathogens *K. pneumoniae* ATCC 10031 and *Enterococcus faecium* ATCC 6569 were used as susceptible strains. Detailed descriptions of the resistant bacterial strains used in the present work (in lab-maintained strains MRSA N315 and MRSA BEC9393; clinically isolated strains VRE 170 and CRE-*Kpn 19*) can be found in the Supplementary Table S1. All bacteria were stored at −20°C or in liquid nitrogen. These strains were deposited in the Microbial Culture Collection of the Microbial Ecology Laboratory, Londrina State University, Brazil.

### Production, Isolation and Identification of Fluopsin C

Fluopsin C production used a patented method (Patent PI0803350-1 – INPI 09/12/20092008)^1^, with some modifications (Bedoya et al., 2019). Briefly, *P. aeruginosa* LV strain was cultured in nutrient broth supplied with 5 mg/L of cooper chloride. After 10 days, the bacterial culture was centrifuged for 20 min at 9000 rpm and 4°C, followed by the extraction of the supernatant with dichloromethane. The extract was purified by flash chromatography with silica gel 60 (0.04–0.062 mm, Macherey-Nagel), coupled to a low-pressure pump and washed with petroleum ether: dichloromethane: Ethyl Ether (65:25:10). A semi-preparative Agilent 1260 Infinity high performance liquid chromatography (HPLC) system, with SB-C18, 4.6 × 250 mm, 5 μm particle size column (Agilent Zorbax Sb-C18), monitored at 264 nm (UV –VIS), was used to purify the Fluopsin C. A gradient of acetonitrile, and water was used as the mobile phase (from 20/80 to 100/0 in 10 min, returning to the original phase for 5 min), with a flow rate of 2 mL.min^–1^ and injection volume of 100 μL. The pure compound was dried and dissolved in deuterated chloroform (CDCI3) or deuterated methanol (CD3OD) at 1,000 μgmL-1. Mass spectra were obtained with an ESI-MS Quattro LCZ (Micromass, Manchester, United Kingdom). ^1^H and ^13^C nuclear magnetic resonance spectra were recorded in solution using a Bruker Avance III 400 MHz spectrometer. X-ray microanalysis (EDS) was carried out using a FEI-Quanta 200 Scanning Electron Microscope with an accelerating voltage of 25 kV. In the experiments, Fluopsin C was reconstituted in DMSO 3%.

### Antibiotic Activity Fluopsin C

The disk-diffusion agar test and minimum inhibitory/bactericidal concentration assays (MIC/MBC) were carried out for evaluating the *in vitro* antibiotic activity of Fluopsin C against different bacteria, in Muller Hinton agar (MHA) and cation-adjusted Muller Hinton broth (MHB), respectively. The tests followed CLSI guidelines (Clinical and Laboratory Standards Institute (CLSI), 2012).

### Resistance Induction and Reversion Experiments

The determination of spontaneous resistant frequency was based on a variation and combination of multi/single passages and mutant prevention concentration (MPC) methods (Nagai et al., 2000; Metzler et al., 2013; Firsov et al., 2015). Briefly, for selecting Fluopsin C-resistant mutants (FC-RMs), serial passages of bacterial strains were performed daily, with MHB containing increasing concentrations of the compound. Initial inocula containing 10^6^ CFU.mL^–1^ were placed into 96-well plates, with 100 μL per well. Fluopsin C was added at concentrations of 0, 0.25×, 0.5×, 1×, 2 × 4×, 8×, and 16× MIC, and plates were incubated at 35°C. Every 24 h, an aliquot of 1 μL was collected from the well treated with the highest concentration of Fluopsin C that presented visible bacterial growth. This aliquot was used to inoculate a fresh micro-broth plate. After 21 days of these sequential passages, the microorganisms from the wells with visible growth, in the highest antibiotic concentration, were distributed in three plates with Fluopsin C-free agar. The MIC and MBC were determined as described before.

For testing the stability of developed resistance, one FC-RM colony was used for serial passages for 10 days, without Fluopsin C, and MIC/MBC were re-evaluated. The remaining colonies were used to inoculate 100 mL of broth, incubated at 35°C for 18 h, under continuous shaking. An aliquot of 100 μL from the bacterial suspension was spread on agar containing 0, 1×, 2×, 4×, 8×, 16× MIC of Fluopsin C. The plates were incubated at 35°C for 48 h. The MPC, the resistance selection frequency in the original strain and their FC-RM clones, as well as their reverted mutant, were determined based on protocols described in literature (Metzler et al., 2013; Firsov et al., 2015).

### Electron Microscopy Analysis

Bacterial suspensions (10^10^ CFU) of the N315 and Kpn19 strains, incubated with and without Fluopsin C, were spotted on polylysine-coated glass slides and kept at 28°C for 1 h for drying. The slides were fixed in a solution of 2% paraformaldehyde and 2.5% glutaraldehyde in 0.1 M sodium cacodylate buffer (pH 7) for 12 h. After fixation, the slides were washed with 0.1 M sodium cacodylate buffer (pH 7) and post-fixed in a 1% OsO_4_ solution for 2 h. The samples were dehydrated in ethanol at concentrations of 70, 80, 90, and 100% and critical-point-dried in CO_2_ (BALTEC CPD 030 Critical Point Dryer). After drying, the slides were coated with gold (BALTEC SDC 050 Sputter Coater) and visualized under scanning electron microscopy (SEM, FEI Quanta 200).

For the transmission electronic microscopy (TEM) assay, microorganisms were incubated with and without Fluopsin C and centrifuged at 4000 rpm for 5 min. The pellets were resuspended and washed with PBS, centrifuged and fixed as described before. After dehydration in a series of ethanol rinses, the material was included in Araldite^®^. Ultrathin cuts of 60–70 nm were collected (Leica Ultracut UCT), contrasted with 2% uranyl acetate (15 min) and lead citrate (20 min), and observed under a transmission electron microscope (FEI Tecnai12).

### *In vitro* Cytotoxicity Assays

The viability of LLC-MK2 cells was determined by the MTT method [dimethylthiazol diphenyl tetrazolium bromide (Sigma Chemical Co., United States)] according to the manufacturer’s recommendation. The antibiotic concentration that inhibited up to 50% of the viable cells, determined by regression analysis, corresponded to the 50% cytotoxic concentration (CC_50__/__24__h_). Human erythrocytes were used for the hemolytic assays.

### Lethal Concentration Assay in Invertebrate Model

*Tenebrio molitor* bugs (Coleoptera) were maintained in our lab as described previously (de Souza et al., 2015). All growth stages were kept together in single plastic containers, with a rearing medium composed of white flour and wheat bran. Bread and vegetable fragments were added periodically. The surface of the culture was covered with filter and water was sprayed daily to provide adequate moisture. The containers were kept in the dark, under controlled environmental conditions (humidity of 70% and temperature at 28°C). *T. molitor* larvae that appeared clear, with uniform color, weighing 150 ± 20 mg, were collected and used in the experiment. For evaluating the lethal concentration of Fluopsin C, groups of larvae (*n* = 10) received intrahemocoelic injections with different drug concentrations (0.06–2 μg per larva). A control group received only sterile diluent (placebo, RPMI with DMSO 3%). Larvae were injected at the ventral surface of the second or third sternite, right above their legs, using a Hamilton syringe. After treatment, all larvae were incubated at 28°C, in Petri dishes containing rearing medium. The treated larvae were observed for 8 days and the following parameters were recorded: melanization of the puncture site, response to a gentle touch stimulus and larvae survival. The experiments were performed with two biological replicates.

### Experimental Murine Model

All mouse experiments were approved by the Animal Care and Use Committee of the State University of Londrina (CEUA – UEL, protocol n°6886.2015.28), and all procedures were in accordance with the standard approved protocols for animal research. The mice were obtained from the UEL Central Animal Facility and acclimatized at the laboratory for at least 48 h. They were kept in polypropylene boxes with wood shaving bedding and placed on ventilated shelves with controlled environmental conditions (24°C, 55% humidity and 12/12 h photoperiod). Sterilized water and commercial feed (Nutival^®^) were provided *ad libitum* throughout the experiment.

Groups of 6 immunocompetent female Swiss albino mice (7 ± 1 week old and 32 ± 3 g), were inoculated intravenously (IV) with 0.1 mL of Fluopsin C in different concentrations (0.5 to 16 mg/kg) or infected intraperitoneally (IP) with 0.5 mL of different concentrations of CRE-Kpn19 strain (10^5^ to 10^9^ UFC/mL), for determining the Lethal Dose (LD) and Lethal Inoculum (LI), respectively. A negative control group was injected with placebo (sterile diluent, RPMI with DMSO 3%) or physiological solution. Mice survival was observed for 48 h.

For the *in vivo* evaluation of the Fluopsin C antimicrobial efficacy against MDR bacteria, groups of 12 randomly assigned mice were inoculated IP with 0.5 ml of CRE-Kpn19 (LI = 4.1 × 10^7^ UFC/mL), and treatments started 2 h post-infection (hpi). Mice received drug injections according to their respective group: (i) control treated with placebo; (ii) a single IV dose of Fluopsin C at 1 mg/kg; (iii) a single IV dose of Fluopsin C at 2 mg/kg; (iv) a single IV dose of Fluopsin C at 3 mg/kg; (v) two IV doses of Fluopsin C at 1 mg/kg, 8 h apart; or (vi) two IV doses of Fluopsin C at 2 mg/kg, 8 h apart. Mortality was recorded over 96 hpi.

### Histopathological Pilot Study

For the evaluation of histopathological toxicity, cohorts of 2 non-infected mice were euthanized at 1, 10, 20, and 40 days after treatment with 2 mg/kg of Fluopsin C or placebo (RPMI medium), both delivered IV. Their whole kidney and liver were collected and fixed in 10% neutral buffered formalin. The organs were dehydrated in ascending series of ethyl alcohol, diaphanized in xylol and included in paraffin. From the paraffin-embedded tissues, 5 μm sections were stained (hematoxylin-eosin) and analyzed qualitatively and quantitatively.

For quantitative analysis, specific portions of the organs were collected and compared using the ImageProPlus software (version 2.5.3). In the liver, the number of hepatocytes with one or two nuclei, hepatocytes with condensed chromatin, and hepatocytes with vacuolization in the cytoplasm were counted. In addition, the area comprising 100 hepatocytes nuclei was measured. In the kidneys, the Bowmann area of ten glomerular corpuscles, the diameter of 50 proximal tubules and 50 distal tubules were measured.

The qualitative analysis was performed by sampling ten random images of liver (400× magnification) and five random images of kidney (100× magnification) of each animal. The presence of inflammatory infiltration, capillary congestion, hemorrhage, cytoplasmic vacuolation, and necrosis was evaluated in both organs. Additionally, the nuclear scaling and position of the cells lining the tubules were observed in the kidney cortex. The severity of such findings was evaluated based on the following scores: 0 - absent, 1 - discrete, 2 - moderate and 3 - severe.

Serum antimicrobial concentrations were determined in seven groups of three healthy Swiss mice, treated with a single IV dose of Fluopsin C (2 mg/Kg). Each group was euthanized at a specific time point after treatment (0, 15, 30, 60, 120, 240, and 480 min post drug injection) and total blood was collected from the mice via cardiac puncture. The blood was immediately placed in microtubes and centrifuged at 3,000 rpm for 5 min at room temperature. The serum was separated and stored at −20°C until analysis. Extraction of Fluopsin C from the serum samples was performed using acetonitrile at low temperature (∼4°C). An internal standard was used for control (500 μg of Phenazine carboxamide (PCN) dissolved in acetonitrile was added to 100 μL of every serum sample before extraction). The sample was vortexed and centrifuged at 3,000 rpm for 5 min at 4°C. The ACN: H_2_O phase was filtered using 13 mm PTFE syringe filters with pore size of 0.22 μm. Fluopsin C was quantified using ultra-pressure liquid chromatography-tandem mass spectrometry (UPLC-MS/MS).

### Statistical Analysis

Statistical analysis and graphics were performed with Rstudio software (2018 RStudio, Inc., - Safari/538.1 Qt/5.4.1). The Kaplan–Meier estimator was applied to generate a survival curve. Statistical analysis was performed using Tukey test, with a confidence level of 95% (significance considered when *p* < 0.05).

## Results

### Identification of Fluopsin C

Fluopsin C was extracted from *P. aeruginosa* LV strain cultures by partitioning their supernatant with dichloromethane and purified by chromatographic adsorption techniques. The fourth fraction obtained by flash chromatography (CF4) was collected and analyzed by HPLC, with about 80% of such fraction corresponding to Fluopsin C. The peak with retention time of 3.611 min was collected and identified using mass spectrometry (265.92 *m/z*), NMR, infrared spectroscopy and X-ray microanalysis (Supplementary Figure S1).

### Antibiotic Activity and Resistance Assays

Fluopsin C presented very strong bactericidal activity against Gram-positive and Gram-negative pathogens, including their MDR variants (Table 1). Moreover, intense bioactivity against *S. aureus, E. faecium*, and *K. pneumoniae* was detected in concentrations below 2 μg/mL.

**TABLE 1:** The Fluopsin C susceptibility tests: disk diffusion on agar (10 μg/disc), MIC, MBC, MPC and mutant frequencies for parent strain and RMs.

**Strain**	**Fluopsin C^†^**	**Halo**	**MIC**	**MBC**	**MPC**	**Mutant frequency**	
		**(mm)**	**(μg/mL)**	**(μg/mL)**	**(μg/mL)**		
						**2xMIC**	**4xMIC**
**MRSA N315**	PS	40	0.5	1.0	4.0	∼4.2 ^∗^ 10^–6^	∼9.2 ^∗^ 10^–9^
	RM21	32	1.0	1.0	4.0	∼2.6 ^∗^ 10^–8^	<1.8 ^∗^ 10^–10^
	RM + 10	39	0.5	1.0	4.0	∼5.0 ^∗^ 10^–7^	<1 ^∗^ 10^–10^
**MRSA BEC9393**	PS	40	1.0	1.0	4.0	∼6.3 ^∗^ 10^–8^	<1.5 ^∗^ 10^–10^
	RM21	38	1.0	1.0	4.0	∼6.5 ^∗^ 10^–7^	<2.5 ^∗^ 10^–10^
	RM + 10	40	1.0	1.0	4.0	∼8.8 ^∗^ 10^–7^	<4.4 ^∗^ 10^–10^
**ATCC 6569**	PS	36	1.0	1.0	4.0	∼4.4 ^∗^ 10^–8^	<6.0 ^∗^ 10^–9^
	RM21	35	1.0	1.0	4.0	∼6.0 ^∗^ 10^–7^	<1.3 ^∗^ 10^–10^
	RM + 10	33	1.0	1.0	4.0	∼9.8 ^∗^ 10^–7^	<2.4 ^∗^ 10^–10^
**VRE-170**	PS	39	1.0	1.0	4.0	∼8.0 ^∗^ 10^–8^	<8.8 ^∗^ 10^–9^
	RM21	28	2.0	2.0	8.0	∼4.3 ^∗^ 10^–6^	<2.5 ^∗^ 10^–10^
	RM + 10	32	1.0	1.0	4.0	∼8.4 ^∗^ 10^–7^	<5.7 ^∗^ 10^–10^
**ATCC 10031**	PS	30	1.0	1.0	4.0	∼3.3 ^∗^ 10^–8^	<1.6 ^∗^ 10^–10^
	RM21	21	4.0	4.0	8.0	∼3.8 ^∗^ 10^–7^	<1.9 ^∗^ 10^–10^
	RM + 10	28	2.0	2.0	4.0	∼6.6 ^∗^ 10^–7^	<3.2 ^∗^ 10^–10^
**CRE *kpn*19**	PS	22	2.0	2.0	8.0	∼4.0 ^∗^ 10^–8^	<3.5 ^∗^ 10^–10^
	RM21	9	16.0	16.0	16.0	<3.5 ^∗^ 10^–10^	<3.5 ^∗^ 10^–10^
	RM + 10	12	4.0	4.0	8.0	∼4.8 ^∗^ 10^–7^	<2.4 ^∗^ 10^–10^

*^†^PS, parent strain; RM21, FC-RM strain after 21 passages of the parent strain on antibiotic-containing media; RM + 10, strain after 21 passages of the parent strain on antibiotic-containing media plus 10 passages on Fluopsin C-free media.*

**FIGURE 1:**
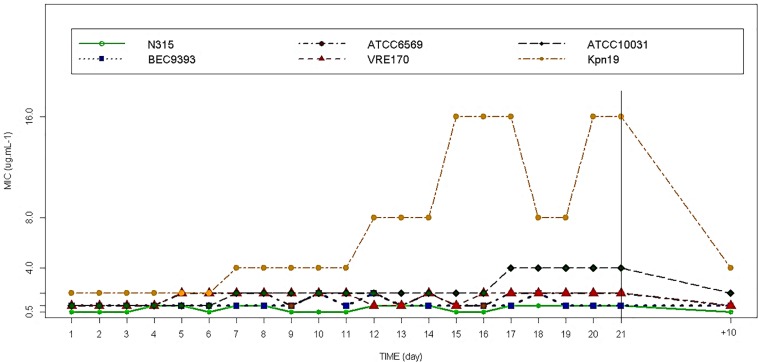
Resistance development of *S. aureus, E. faecium* and *K. pneumoniae* during 21 serial passages in Fluopsin C-containing MHB (FC-RM21) and the final MIC, when the mutants were serially passaged for 10 days in Fluopsin C-free medium (FC-RM + 10).

The generation of Fluopsin C-resistant mutants was tested with bacteria cultured in sub-MIC concentrations for 21 days (FC-RM21), as shown in Figure 1. Serial passaging of *K. pneumoniae* (ATCC 10031 and *Kpn-*KPC 19) cultures containing Fluopsin C led to a moderate loss in susceptibility, with Fluopsin C-MIC gradually increasing to 4-fold and 8-fold after the 21st passage, for ATCC 10031 and *Kpn-*KPC 19, respectively. The MIC decreased again when the mutants were serially passaged for 10 days in Fluopsin C-free culture (FC-RM + 10), indicating a tendency for bacterial resistance reversal and consequent return to the original MIC. No resistant mutants of *S. aureus* and *E. faecium* were detected after 21 days of serial passages in different concentrations of Fluopsin C.

**FIGURE 2:**
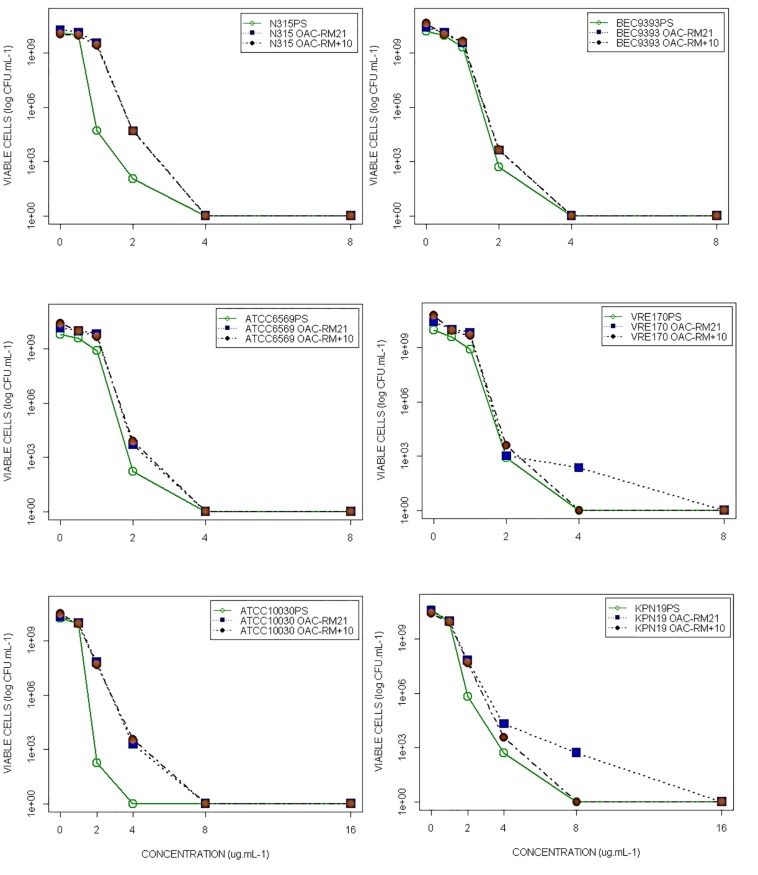
Fluopsin C-MPC determination with *S. aureus* (MRSA N315 and MRSA BEC9393), *E. faecium* (ATCC6569 and VRE 170) and *K. pneumoniae* (ATCC 10031 and CRE-Kpn19) parent and FC-RMs strains.

Results from the susceptibility tests (i.e., disk diffusion (10 μg/disk), MIC and MBC), using FC-RM21 and FC-RM + 10, are presented in Table 1. The MPC was determined as the antibiotic concentration necessary to prevent the growth of FC-RMs on Fluopsin C-containing agar plates after recovering ∼10^10^ CFU from the cell suspension culture. Following 48 h, no resistant colonies were observed, and all strains tested were unable to produce resistant mutants, when plating on media with Fluopsin C (4× MIC), giving the calculated frequency of resistance below 10^–9^. The frequency of resistant clones cultured in 2× MIC of Fluopsin C was determined as well. The MPC and frequencies are shown in Table 1 and Figure 2.

### Ultrastructural Analysis

**FIGURE 3:**
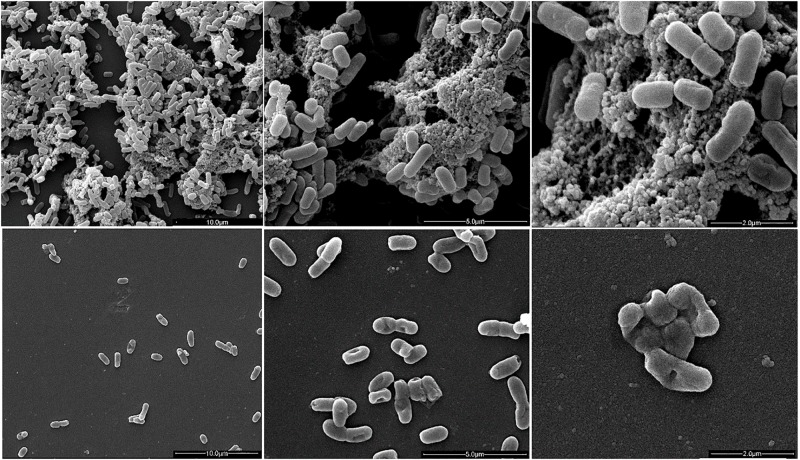
Scanning electron micrographs (SEM). Above, control (not treated), large number of *K. pneumoniae* with intact appearance and high extracellular polysaccharide production. Below, *K. pneumoniae* treated with Fluopsin C (1 h), showing fewer microorganisms and bacteria presenting cell wall disruption, forming depressions in the configuration of the bacterial skeleton and lessened extracellular matrix.

**FIGURE 4:**
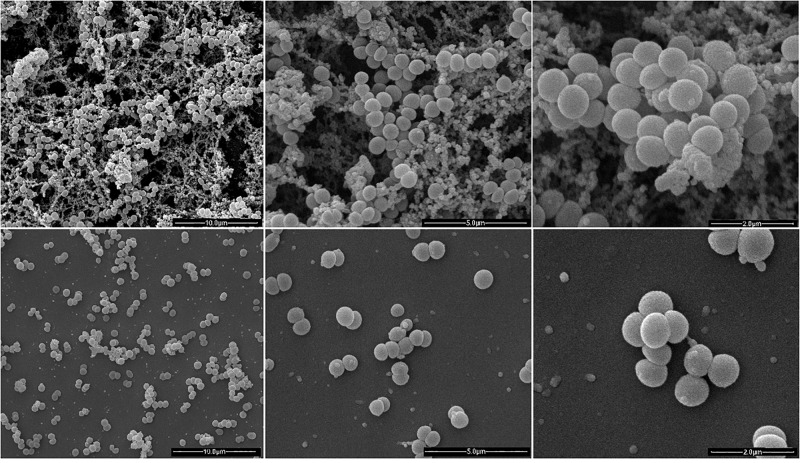
Scanning electron micrographs (SEM). Above, MRSA-N315 control, great number of bacteria with intact appearance and high extracellular polysaccharide production. Below, MRSA-N315 strain treated with Fluopsin C (1 h), showing less bacteria and extracellular matrix.

**FIGURE 5:**
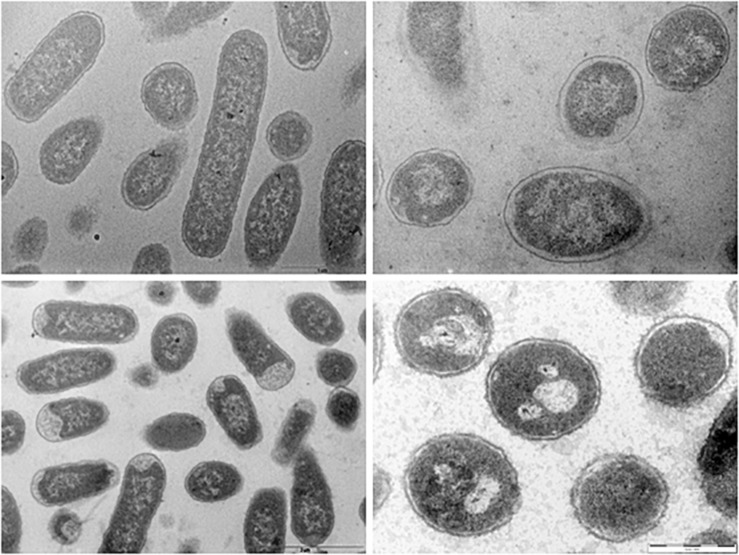
Transmission electron micrographs. Control (above) and Fluopsin C-treated *K. pneumoniae* cells (below). TEM studies demonstrated that Fluopsin C induced internal cell damage.

**FIGURE 6:**
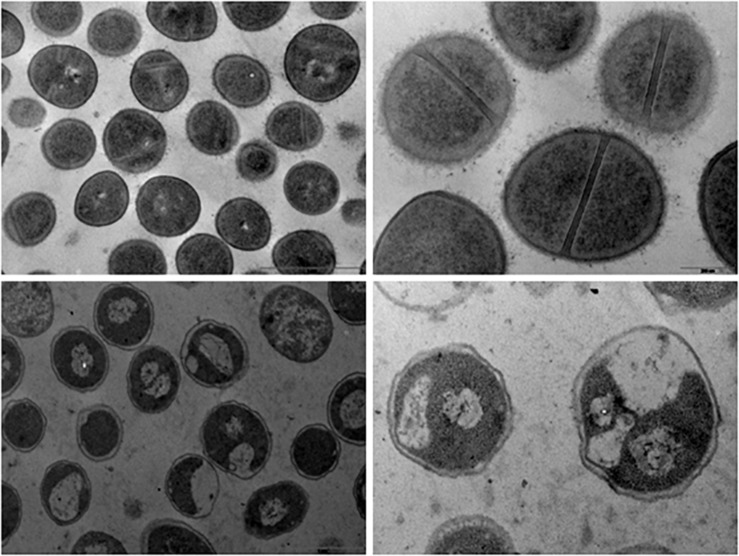
Transmission electron micrographs. Control (above) and Fluopsin C-treated MRSA cells (below). TEM studies demonstrated that Fluopsin C induced internal cell damage.

The ultrastructure of Fluopsin C-treated cells was evaluated by Scanning Electron Microscopy (SEM) and Transmission Electron Microscopy (TEM), after 1 h of incubation. Clinical isolates of *K. pneumoniae* (*Kpn*-KPC 19) and MRSA (N315) incubated with Fluopsin C presented reduced CFU and decreased extracellular matrix formation when compared to bacteria incubated without the compound (Figures 3, 4). Fluopsin C decreased the number of bacteria and generated morphological changes in bacterial cell shape, with marked depressions in the cell shape, lessened extracellular matrix and visible cell disruption (Figures 3, 4). Cytoplasmic alteration and vacuolation were detected by TEM in cells treated with Fluopsin C when compared to non-treated cells (Figures 5, 6). The plasma membrane was intact, but the cell showed low electron density, probably caused by a failure in the ionic pumps of the plasma membrane.

### *In vitro* and *in vivo* (Invertebrate Model) Toxicity Evaluation

The cytotoxic and hemolytic effects analyses were carried out using LLC-MK2 cells and human erythrocytes, respectively. Fluopsin C induced the highest toxic activities (>90%) with ≥20 μg/mL. The lowest toxic concentration (CC_50__/__24__h_) detected in both LLC-MK2 and red blood cells was near 2 μg/mL. When Fluopsin C concentration was below 2 μg/mL, no cytotoxic or hemolytic effect was observed (Supplementary Figure S2). Larvae of *T. molitor* treated with 0.06 and 0.12 μg of Fluopsin C, as well as control larvae, did not indicate any toxic effect. Larva groups treated with 0.25 and 0.5 μg presented mortality rates of 20 and 42%, respectively, whereas all larvae treated with 1 and 2 μg died after 96 hpi (Supplementary Figure S3A).

### Antibiotic Activity Evaluation in Mice Model

Fluopsin C demonstrated strong antibiotic activity against *kpn*-KPC 19 strain in *in vitro* test (MIC = 2 μg/mL), as described above. Also, this concentration did not generate evident toxic effects. After these *in vitro* results were obtained, the effect of Fluopsin C for controlling *kpn*-KPC 19 experimental peritoneal infection of mice was evaluated. Initially, the lethal dose (LD) of Fluopsin C was determined, where concentrations below 4 mg/kg (LD_50_) did not generate toxicity signs in treated mice after 96 h. However, higher doses produced lethality after 48 h (Supplementary Figure S3B). The presence of Fluopsin C in serum was assessed by UPLC-MS/MS, without detection in any time point.

**FIGURE 7:**
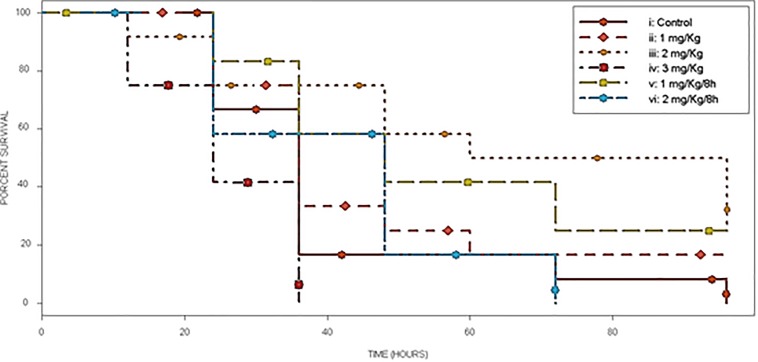
Survival curve of the experimental murine model of MDR infection. Control (i) and 5 different treatments (ii–vi) with Fluopsin C, in septicemia protection model using CRE–Kpn19. Survival is depicted until 96 h after infection.

The *in vivo* evaluation of Fluopsin C antibiotic activity was performed using a murine sepsis model. Swiss mice were infected by IP route with a lethal dose (LI_90_) of *kpn*-KPC 19 cell suspension, being treated with Fluopsin C or placebo after infection. Mice treated with a single dose of 2 mg/kg and two doses of 1 mg/kg 8 h apart (experimental groups iii and v, respectively) presented survival rates of 50 and 42%, respectively, after 72 h of treatment. The group treated with one dose of 1 mg/kg (treatment ii) showed a survival rate of 25% after 48 h and 17% after 96 h. Groups iv and vi (3 mg/kg and two doses of 2 mg/kg/8 h, respectively) presented lower survival rates, reaching complete mortality even faster than control group (Figure 7).

### Histopathological Examination

**FIGURE 8:**
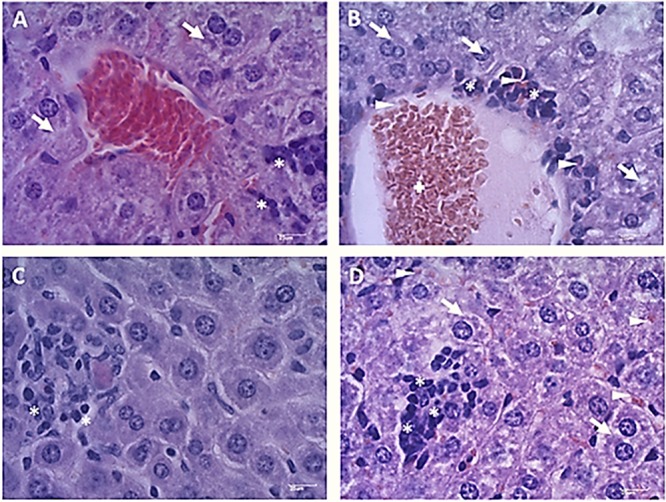
Photomicrographs of mice liver 10 days after treatment with Fluopsin C or placebo. The control group **(A,C)** showed a slight cytoplasmic vacuolation of hepatocytes (arrow) and discrete inflammatory infiltrate (asterisk), while animals treated with Fluopsin C **(B,D**) presented moderate vessel congestion (cross), marked vacuolation of the cytoplasm (arrow), moderate inflammatory infiltrate (asterisk) and bleeding (arrowhead). Similar images were observed in other fields, in every time point. Color: HE. Scale bar: 25 μm.

Liver and kidney of mice were collected at 1, 10, 20, and 40 days after treatment with Fluopsin C and placebo. The control group did not show overt alterations in the morphology of hepatic cells, but areas with discrete hepatocyte cytoplasm vacuolation and inflammatory infiltration were detected (Figure 8). Liver collected from mice treated with 2 mg/kg of Fluopsin C presented moderate vessel congestion, inflammatory infiltration, hemorrhage and marked cytoplasmic vacuolation in all time points, but there was no sign of necrosis during the whole experiment (Supplementary Table S2). Fluopsin C did not cause significant changes in the number of hepatocytes, neither with one or two nuclei, nor with condensed chromatin. Mild differences of hepatocyte nuclear area were observed in both groups, in all time points. Hepatocytes with vacuolated cytoplasm and displaced nucleus were detected in treated mice (Supplementary Table S3 and Supplementary Figure S4).

**FIGURE 9:**
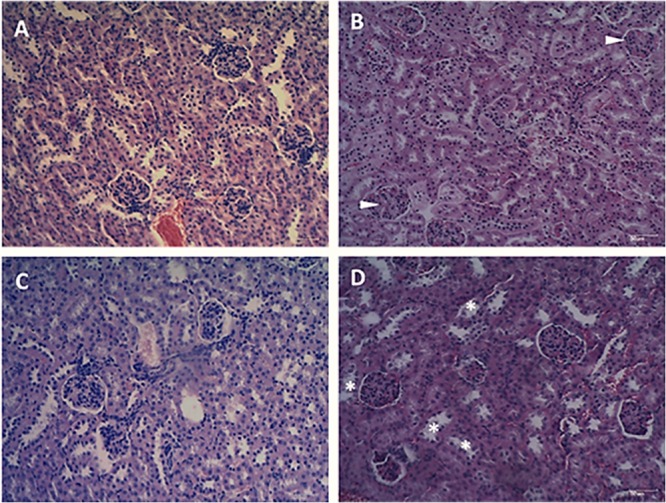
Photomicrographs of the renal cortex of mice after 10 days of treatment with Fluopsin C or placebo. The control group **(A,C)** presented mild inflammatory infiltrate and bleeding in the glomerular corpuscles. In the tubules **(C)**, discrete cytoplasmic vacuolation could be observed and scaling was practically non-existent. The animals in the treated group **(B,D)** presented no glomerular changes (arrowheads), but a mild peeling of the tubular epithelium (asterisk) was detected. Similar images were observed in other fields and on other days. Color: HE. Scale bar: 25 μm.

The qualitative analysis of kidney did not detect congestion, edema or necrosis in glomerular corpuscles (Figure 9). However, mild inflammatory infiltration and bleeding was present in treated mice. In the tubules, slight cytoplasmic vacuolation and nuclei positioned at the cell center were observed. Differences in the number of glomerular corpuscles, changes in the Bowman’s space and tubule diameters were observed (Supplementary Table S4). No significant differences were observed between the renal cortexes of mice treated with Fluopsin C and placebo (Supplementary Figure S4).

## Discussion

Current treatments of MDR infections are often unsuccessful due to continuous selection of bacteria resistant to a large number of antibiotics. It seems that the biggest challenge now is to discover novel drug candidates that may overcome bacterial resistance mechanisms. Previous studies have reported the antibiotic effects of secondary metabolites of *Pseudomonas* sp. against many clinical pathogenic resistant microorganisms. The F3d fraction, produced by *P. aeruginosa* LV strain, demonstrated activity against N315, BEC9393, ATCC 10031, and *Kpn*-KPC 19 strains (Cardozo et al., 2013; Kerbauy et al., 2016), with the metalloantibiotic compound Fluopsin C being the major component with strong antibiotic activity (de Oliveira et al., 2016). Elementary analysis, mass spectrometry, chemical proprieties and biological activity evaluation proved that *P. aeruginosa* LV strain-metalloantibiotic is identical to Fluopsin C (YC 73), a compound described in earlier studies (Egawa et al., 1970, 1971; Itoh et al., 1970; Otsuka et al., 1972; Ma et al., 2013; de Oliveira et al., 2016).

Fluopsin C demonstrated a potential increase of 250-fold in biocidal activity when compared to the F3d fraction, and it required 50-fold less concentration to form equal or greater inhibition halos than the ones described in previous experiments with less purified fractions (Cardozo et al., 2013; Kerbauy et al., 2016). In addition, Fluopsin C decreased MIC 30- and 125-fold when tested against *K. pneumoniae* and *S. aureus*, respectively (Cardozo et al., 2013; Kerbauy et al., 2016). Fluopsin C was effective against Gram-positive and Gram-negative bacteria, with MICs comparable to commercial antibiotics commonly used in clinical practice, such as vancomycin, and new antibiotics, such as linezolid, teixobactin and daptomycin (Firsov et al., 2015; Ling et al., 2015). The bactericidal activity of Fluopsin C was analyzed under electron microscopy, allowing the comparison between treated and non-treated bacterial cultures. In both SEM and TEM it was possible to observe that Fluopsin C caused cell lysis and degraded cellular matrix. Similar effects were observed when cells were treated with the semi-purified F3d fraction (Cardozo et al., 2013; Kerbauy et al., 2016). The present results raised the hypothesis that the primary target of Fluopsin C is the cell membrane, causing its disruption, but further studies need to be carried to better understand the action of Fluopsin C in the cell.

Previous studies using serial passages selected resistant mutants of *K. pneumoniae, S. aureus, Escherichia coli*, and *Streptococcus pneumoniae* for different antibiotics, such as Fluoroquinolones, Linezolid, Daptomycin and Vancomycin. Their respective MIC increased 100 times in a few days when compared to the original strain (Nagai et al., 2000; Firsov et al., 2006, 2015; Ling et al., 2015; Strukova et al., 2017). In the present study, FC-RMs did not generate spontaneous resistant mutants of Gram-positive bacteria during 21 serial passages with sub-inhibitory concentrations of Fluopsin C. On the other hand, *K. pneumoniae* ATCC 10031 strain produced FC-RM21, increasing the MIC 4-fold, but the resistance stability was affected at the 10th transfer to antibiotic-free media, when the original MIC was restored. The results indicated that Fluopsin C-tolerant clones of *K. pneumoniae* were obtained during the multi-passages, when the antibiotic was present, but the MIC increased below the level detected in previous studies with other antibiotics (Nagai et al., 2000; Firsov et al., 2006, 2015; Ling et al., 2015; Strukova et al., 2017). The *kpn*-KPC 19 produced after 21 passages (FC-RM21) increased MIC eight-fold (2 to 16 μg/mL) and the MPC only twofold (8 to 16 μg/mL), suggesting that it is hard for bacteria to generate resistance to Fluopsin C in concentrations above 16 μg/mL. Furthermore, the resistance frequency to Fluopsin C was low, with CFU below 10^–9^. The MIC of *kpn*-KPC 19 possibly increased by intrinsic resistance mechanisms, such as efflux pumps, which could transport Fluopsin C to outside the cell, but further molecular studies should be made to determine which defense mechanisms are involved.

The methods applied in this study emphasized the importance of evaluating the emergence of microbial resistance to new antibiotic molecules, with little expenses. Molecular techniques (Martínez et al., 2011; Andersson, 2015) and dynamic models of “anti-mutant” ratios (Firsov et al., 2015; Strukova et al., 2017; Yu and Wang, 2017) are also important methods that can improve the prediction of mutational resistance rise to antimicrobial substances, even before their clinical use. Current antibiotic treatments for *K. pneumoniae* infections are often unsuccessful due to the increasing frequency of antibiotic resistance genes, which creates MDR strains, and the low number of drug candidates in clinical development. *P. aeruginosa* secondary metabolites, expressed in culture medium with copper, were used to identify a class of organic metal complexes with potent antibiotic activity. Fluopsin C demonstrated very strong biocidal activity in *in vitro* experiments against *K. pneumoniae*, including many resistant strains with different resistance genes (Kerbauy et al., 2016). Beyond that, this compound was active against Gram-positive bacteria, including clinical isolates of *S. aureus* MRSA and *E. faecium* VRE (Cardozo et al., 2013).

The chemotherapeutic potential of Fluopsin C has been highlighted since its first description, but the significant cytotoxicity against Ehrlich, Hela, and sarcoma 180 cells line, added to acute toxicity in animal models, suggested its unfeasibility to control infections in humans (Itoh et al., 1970; Otsuka et al., 1972). Fluopsin C also increased CC_50__/__24 h_ when compared to previous studies with semi-purified fractions, where the cytotoxicity was not detected with F3 and F3d fractions (Murate et al., 2015; Kerbauy et al., 2016). Probably, the activity of these fractions against LLCMK2 cell line was low because Fluopsin C was diluted in these fractions. The replacement of live animals by alternative models is desirable and studies for measuring bioactivity using invertebrate larvae increase each year (Desbois and Coote, 2011; Luther et al., 2014; de Souza et al., 2015). The *T. molitor* larva model indicated a LD_50_ of 0.5 μg per larvae (∼3 mg/kg), very close to the result found in the murine model (LD_50_ of 4 mg/kg), thus supporting the application of such a model and the reduction in the use of animals in the experiment.

The low pharmacokinetic properties can be an issue to most natural products (Das et al., 2016). From the best of our knowledge, there is no previous report of Fluopsin C detection in blood. In the present study, the compound could not be detected in the blood of treated mice, suggesting a low bioavailability. Despite that, the metalloantibiotic significantly increased the survival rate of Swiss mice infected with *kpn-*KPC 19, protecting a high percentage of the infected animals against sepsis. It is possible that Fluopsin C is quickly metabolized by the treated organism and changed into a different molecule, or molecules, with distinct chemical properties, complicating its detection. Another possibility is that the compound may have such high affinity for the membrane cells, that it might be trapped on them. Still, further studies must be carried out to verify such hypotheses.

This is the first report of Fluopsin C efficacy in MDR-infected mice. In addition, Fluopsin C did not cause pathological alterations in kidney cells, such as glomerular corpuscles and the Bowman’s space area, suggesting that Fluopsin C is not a nephrotoxic compound. On the other hand, the treatment increased the frequency of hepatocytes with vacuolated cytoplasm and displaced nucleus. These changes occur when triglyceride production increases during liver damage, suggesting that the antibiotic presents moderate hepatotoxicity (Ma et al., 2013). Likewise, many other antibiotics, such as isoniazid, rifampicin, and pyrazinamide, commonly used in clinical therapy, cause moderate liver damage during the treatment of tuberculosis patients, but they are still applied in the control of infection (Rossouw and Saunders, 1975). Vancomycin is indicated to treat Gram-positive bacteria, but it is not considered a first-choice drug because of the common adverse effects, like hepatotoxicity, nephrotoxicity and phlebotoxicity (Bruniera et al., 2014). In the same way, the intravenous formulation of colistin and polymyxin B were gradually abandoned in many countries in the early 1980s because of the high incidence of severe nephrotoxicity. However, due to the emergence of MDR in most of the antibiotic classes available and the lack of new antimicrobial against Gram-negative, the polymyxins were put back in use as a therapeutic option (Falagas and Kasiakou, 2005). The present study provides new information about the potent antibiotic activity of Fluopsin C against MDR isolates. Still, its toxicity and antibiotic selectivity should be better studied. Thus, despite the small gap between effective healing and toxic doses, Fluopsin C, or a possible derivative, may become a new member in the antibacterial arsenal against MDR infections.

## Conclusion

The strong antibiotic activity of Fluopsin C under *in vitro* and *in vivo* conditions stressed the potential of this compound to control Gram-positive and Gram-negative MDR infections. However, the development of a less cytotoxic derivate or presentation is desirable. Additional studies aiming at the reduction of toxicity – by reducing the Fluopsin C effective concentration with possible synergic combinations with commercial antibiotics – as well as the elucidation of its pharmacokinetic properties and mechanisms of action will be carried out in the near future.

## Data Availability Statement

All datasets generated for this study are included in the manuscript/Supplementary Files.

## Ethics Statement

All animal experimentation was carried out with the approval of the Animal Research Ethics Committee of the State University of Londrina (CEUA – UEL, protocol n°176;6886.2015.28) and all procedures were in accordance with the standard approved protocols for animal research and approved by the name of committee. The arrive checklist can be found in Supplementary Material.

## Author Contributions

GA and MN conceived the study and designed the experimental procedures. MN, AS, JP, AB, JE, EN, MA, FM, MD, TC, and OS carried out the experiments. MN, EA, DS, AC, and GA analyzed the data. EA, DS, and PB contributed the reagents and materials. MN and AC wrote the manuscript. GA supervised the project.

## Conflict of Interest

The authors declare that the research was conducted in the absence of any commercial or financial relationships that could be construed as a potential conflict of interest.

## Footnotes

1 http://www.inpi.gov.br
