# Supplementary material for: Fluopsin C for Treating Multidrug-Resistant Infections: In vitro Activity Against Clinically Important Strains and in vivo Efficacy Against Carbapenemase-Producing Klebsiella pneumoniae
Source: Front Microbiol. 2019 Oct 25;10:2431. doi: 10.3389/fmicb.2019.02431 (PMC6824035; doi:10.3389/fmicb.2019.02431)
Supplement: Supplementary file 1 [file Table_1.DOCX]

Supplementary Material

# Supplementary Data

**Table S-1. Multidrug resistant bacteria used in this study.**

| **Strain** |  | **Phenotypic Antibiotic Resistance** |  | **Precedence/Reference** |
| --- | --- | --- | --- | --- |
| **MRSA N315** |  | β-lactams |  | Japan patient[1] |
| **MRSA BEC9393** |  | β-lactams |  | Brazilian Epidemic Clone[2] |
| **VRE 170** |  | β-lactams, Vancomycin |  | Isolated University Hospital of Londrina [3] |
| **CRE-*Kpn 19*** |  | Imipenem, meropenem, ertapenem, polimixin B, colistin |  | Isolated University Hospital of Londrina [3] |

**Table S-2.** Qualitative analysis of the liver of mice at 1, 10, 20 and 40 days after treatment with 2mg/Kg of fluopsin C (T) or placebo (C).

| **PARAMETER** | **SCORE** | | | | | | | |
| --- | --- | --- | --- | --- | --- | --- | --- | --- |
|  | **C1** | **T1** | **C10** | **T10** | **C20** | **T20** | **C40** | **T40** |
| **vessel congestion** | 0 | 1 | 0 | 3 | 0 | 2 | 0 | 2 |
| **cytoplasmic vacuolization** | 1 | 1 | 1 | 2 | 1 | 3 | 1 | 3 |
| **inflammatory infiltrate** | 1 | 2 | 1 | 2 | 1 | 1 | 1 | 2 |
| **Necrosis** | 0 | 0 | 0 | 0 | 0 | 0 | 0 | 0 |
| **hemorrhage** | 0 | 3 | 0 | 0 | 0 | 2 | 0 | 2 |

scores: 0 - absent, 1 - discrete, 2 - moderate and 3 – accented

**Table S-3.** Quantification of hepatocytes with vacuolated cytoplasm and displaced nucleus (10 fields) of mice at 1, 10, 20 and 40 days after treatment with 2mg/Kg of fluopsin C (T) or placebo (C).

| **HEPATOCYTES WITH** | **QUANTIFICATION** | | | | | | | |
| --- | --- | --- | --- | --- | --- | --- | --- | --- |
|  | **C1** | **T1** | **C10** | **T10** | **C20** | **T20** | **C40** | **T40** |
| **1 nuclei** | 229 | 202 | 268 | 246 | 253 | 189 | 333 | 170 |
| **2 nucleus** | 15 | 11 | 17 | 47 | 16 | 33 | 36 | 42 |
| **Condensed Chromatin** | 35 | 40 | 28 | 48 | 35 | 8 | 39 | 45 |
| **Vacuolization** | 1 | 207 | 5 | 115 | 8 | 226 | 3 | 206 |

**Table S-4.** Qualitative analysis of the kidney of mice at 1, 10, 20 and 40 days after treatment with 2mg/Kg of fluopsin C (T) or placebo (C).

| **PARAMETER** | **SCORE** | | | | | | | |
| --- | --- | --- | --- | --- | --- | --- | --- | --- |
|  | **C1** | **T1** | **C10** | **T10** | **C20** | **T20** | **C40** | **T40** |
| **Congestion of capillaries** | 0 | 0 | 0 | 0 | 0 | 0 | 0 | 0 |
| **Edema** | 0 | 0 | 0 | 0 | 0 | 0 | 0 | 0 |
| **Necrosis** | 0 | 0 | 0 | 0 | 0 | 0 | 0 | 0 |
| **Inflammatory infiltrate** | 1 | 1 | 1 | 1 | 1 | 1 | 1 | 1 |
| **Bleeding** | 1 | 1 | 1 | 1 | 1 | 1 | 1 | 1 |
| **Vacuolisation of the cytoplasm** | 1 | 2 | 1 | 1 | 1 | 1 | 1 | 3 |
| **Desquamation** | 0 | 1 | 1 | 1 | 0 | 1 | 1 | 1 |
| **Core position** | M* | M | M | M | M | M | M | M |

scores: 0 - absent, 1 - discrete, 2 - moderate and 3 – accented

*Medial


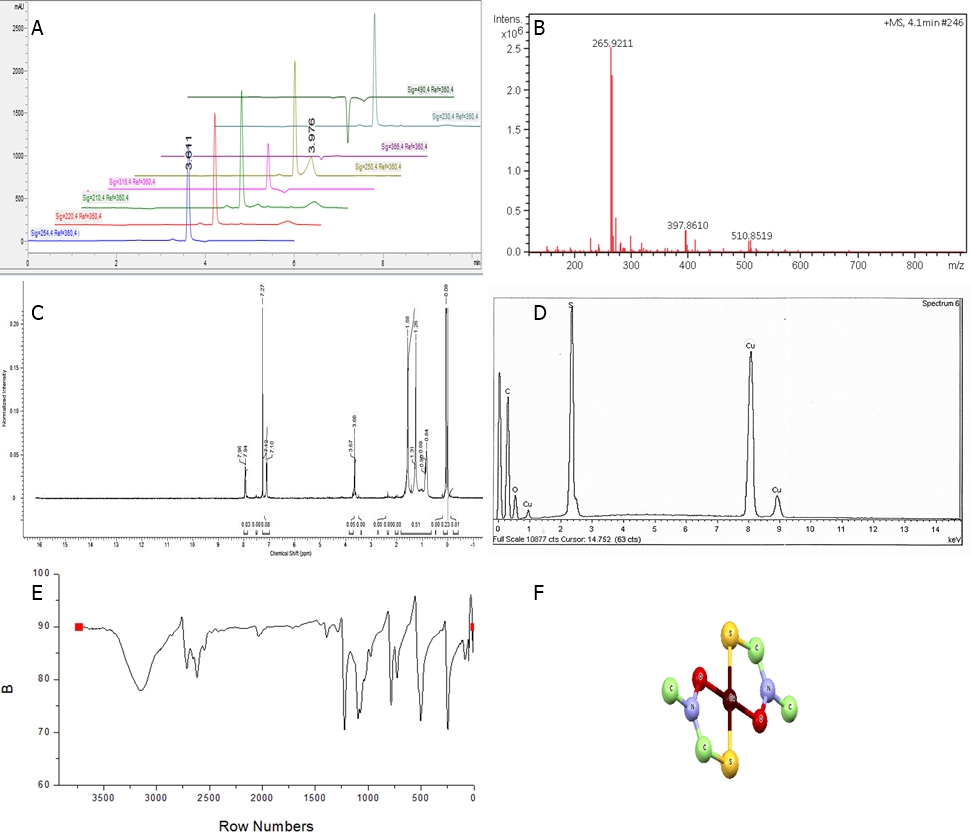


**Supplementary Figure S-1. A)** HPLC chromatogram of CF4 fraction in different wavelengths (264, 220, 210, 316, 250, 366, 230, 290 nm, respectively). The peak with retention time of 3.611 min was quantified (R^2^ = 0.999) and collected, **B)** ESI-MS (positive mode). Mass = 265.92 *m/z*, **C)** ^1^H NMR of OAC (300 MHz; CDCl_3_), **D)** SEM - EDS spectrum of OAC, **E)** Infra-Red of the natural organocopper antibiotic compound (OAC) produced by *Pseudomonas aeruginosa* LV strain. **F)** Chemical structure of OAC identified as fluopsin C.


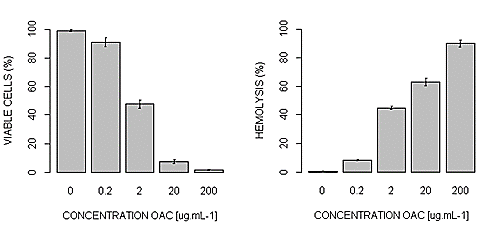


**Supplementary Figure S-2***.* Cytotoxicity and Hemolytic analysis. Cells after 24 h of treatment with different concentrations of fluopsin C.


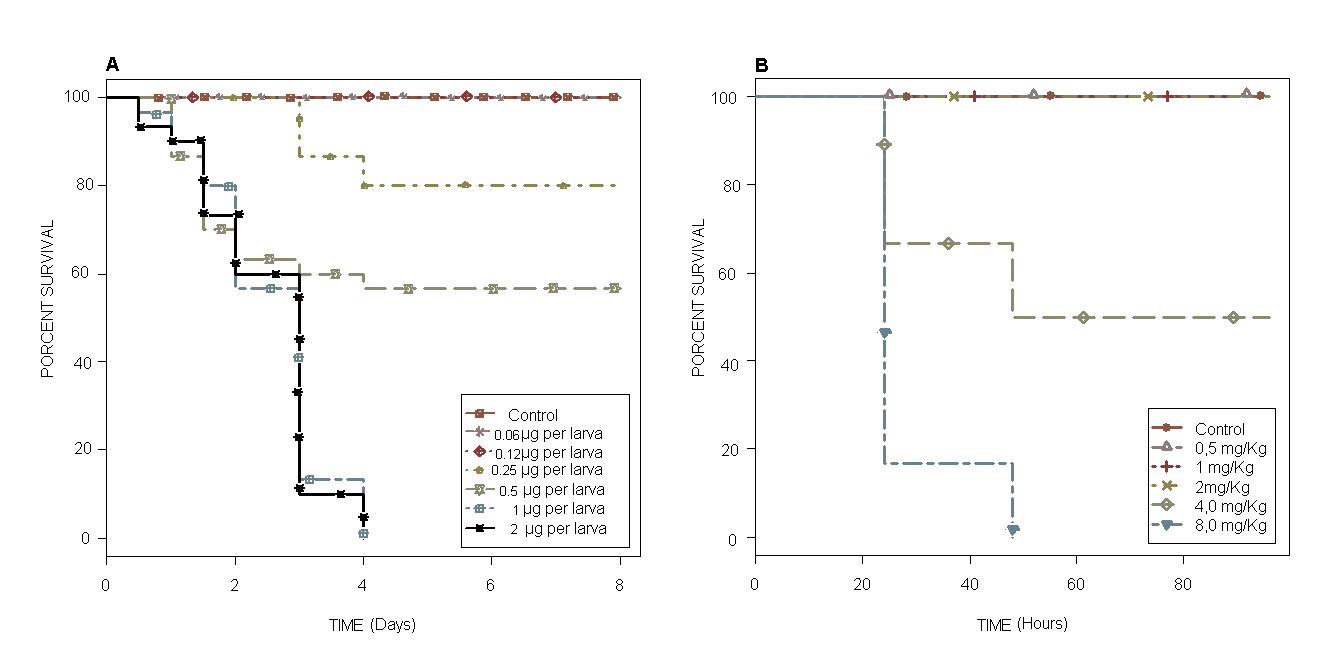


**Supplementary Figure S-3.** Effect of antibiotic on survival of Tenebrio molitor larvae (A) and mice (B). Treatment with different concentrations of fluopsin C in 8 days and 96 hours of observation, respectively.


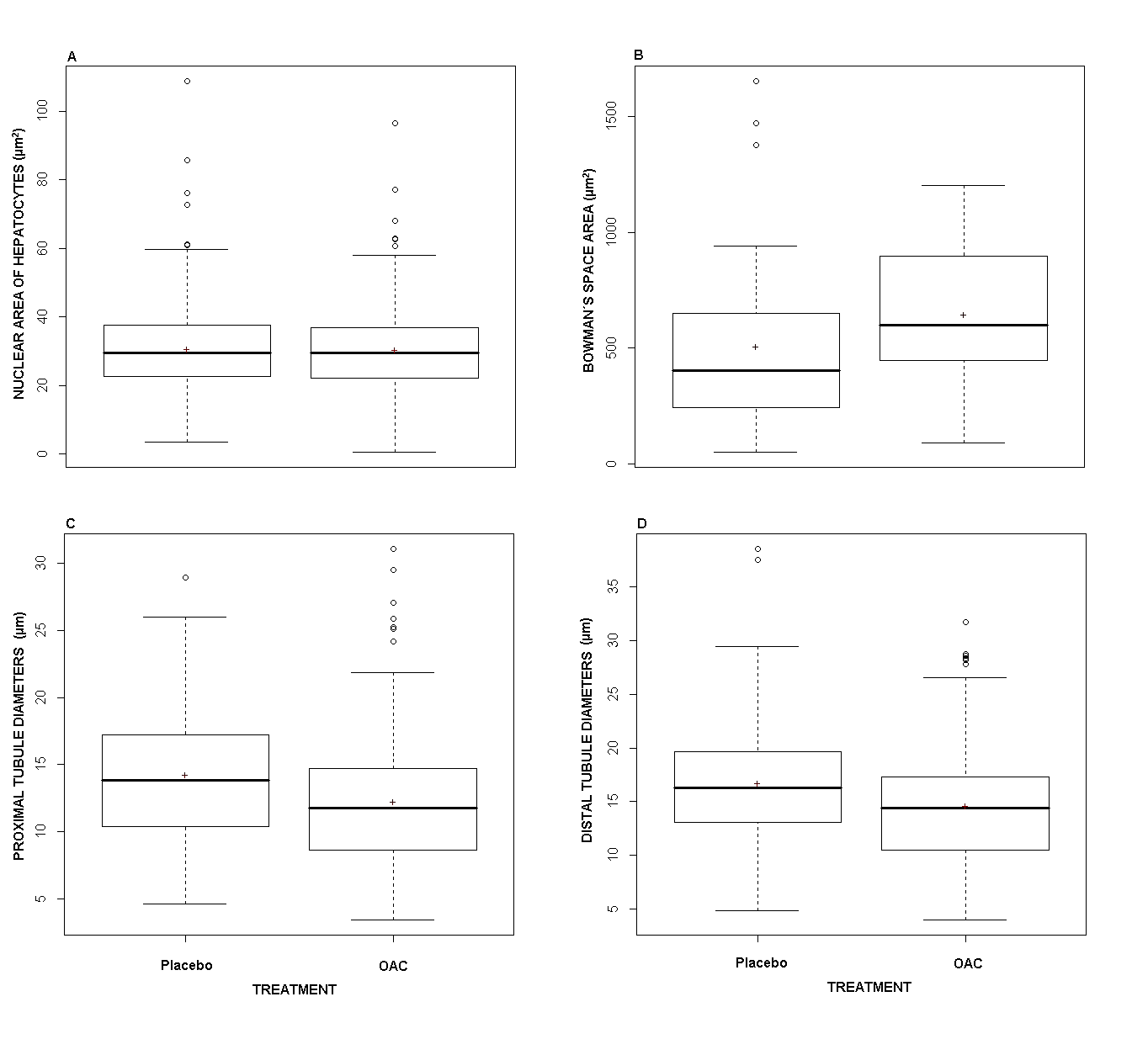


**Figure S-4.** Box plots. (A) Area of 400 nucleus of hepatocytes, (B) area of 400 Bowman´s space, (C) diameters of 200 proximal tubule and (D) diameters of 200 distal tubule. The median (-) and mean (+) of each treatment for the sums of the all times.
