# Supplementary material for: Fluopsin C for Treating Multidrug-Resistant Infections: In vitro Activity Against Clinically Important Strains and in vivo Efficacy Against Carbapenemase-Producing Klebsiella pneumoniae
Source: Front Microbiol. 2019 Oct 25;10:2431. doi: 10.3389/fmicb.2019.02431 (PMC6824035; doi:10.3389/fmicb.2019.02431)
Supplement: Supplementary file 2 [file Table_2.DOC]

**THE ARRIVE Guidelines Checklist.**

| Topic | ITEM  No. | RECOMMENDATION | Section/ Paragraph |
| --- | --- | --- | --- |
| **Title** | 1 | **Fluopsin C for Treating Multidrug-Resistant Infections: *In Vitro* Activities against Clinical Important Strains and *In Vivo* efficacy against *Klebsiella pneumoniae* Carbapenemase (KPC)-producing *K. pneumoniae*.** | Title |
| **Abstract** | 2 | To evaluate *in vitro* and *in vivo* activities of fluopsin C against Multidrug-Resistant bacteria. The bioactivity, resistance-development risk, toxicity and therapeutic efficacy of fluopsin C using a murine model of sepsis, as well as post-treatment histopathological analysis, were determined.  The therapeutic efficacy of intravenous fluopsin C administration was evaluated in a murine model of *Klebsiella pneumoniae* (KPC) acute sepsis, using six different treatments.  Best therapeutic results were achieved in mice treated with a single dose of 2 mg/kg and in mice treated with two doses of 1 mg/Kg, 8 hours apart. Furthermore, acute and chronic histopathological studies demonstrated absent nephrotoxicity and moderate hepatotoxicity.  The results demonstrated the efficacy of fluopsin C against MDR organisms, in *in vitro* and *in vivo* models, and hence it can be a novel therapeutic agent for the control of MDR infections in severe cases. | Abstract |
| **Introduction** |  |  |  |
| **Background** | 3 | The antimicrobial resistance (AMR), which includes multidrug resistance (MDR), is the main obstacle to tackle in order to increase successful treatment rates and lower death numbers of severe hospital-acquired infections. The discovery and development of novel antibiotic molecules and compounds, with high activity against untreatable MDR bacteria, is utterly necessary.^1^  Previous studies demonstrated that specific fractions obtained from the culture of *P. aeruginosa* LV strain (F3, F3D and F4A) present potent antimicrobial activity, against many pathogens. ^6–12^ However, there is a lack of studies on microbial resistance development, ultrastructural effect in blank pathogens and *in vivo* efficacy of fluopsin C, which may confirm the possibility of using this compound as an alternative for the treatment of severe human infections.  New assays to evaluate the bioactivity, resistance-development risk, toxicity and therapeutic efficacy of fluopsin C, as well as post-treatment histopathological analysis, are required to determine the suitability of its therapeutic application. | Introduction |
| **Objectives** | 4 | The aim of the present study was to evaluate *in vitro* and *in vivo* activities of fluopsin C against MDR bacteria. To determine the haemolytic and cytotoxic effects of fluopsin C, we used blood and mammalian cells culture. *Tenebrio molitor* larvae were used to evaluate fluopsin C lethal concentration, before the determination of therapeutic efficacy against *K. pneumonia* (KPC) in a murine sepsis model. The kidney and liver histopathological alterations were also studied. | Introduction / Line 80 |
| **Methods** |  |  |  |
| **Ethical statement** | 5 | All mouse experiments were in accordance and approved by the Animal Care and Use Committee of the State University of Londrina - UEL (protocol n°6886.2015.28). | Experimental Murine Model |
| **Study design** | 6 | Groups of 6 mice were injected with fluopsin Cor CRE-Kpn19 strain, for determining the Lethal Dose (LD) or Lethal Inoculum (LI), respectively. For efficacy experimental design, groups of 12 randomly assigned mice were used in six different treatments. For the histopathological analysis, cohorts of 2 mice were euthanized for each time point. | Experimental Murine Model |
| **Experimental procedures** | 7 | Groups of 6 mice were inoculated intravenously (IV) with 0.1 mL of different doses (0.5 – 16 mg/Kg) of fluopsin C or infected intraperitoneally (IP) with 0.5 mL of different concentrations (10^5^ to 10^9^ UFC/mL) of CRE-Kpn19 strain, for determining the Lethal Dose (LD) and Lethal Inoculum (LI), respectively. A negative control group was injected with physiological solution. Mice survival was observed for 48 h.  For the evaluation of the therapeutic antimicrobial action of fluopsin C against MDR bacteria, experimental groups of 12 randomly assigned mice were used. The animals received an IP inoculum of 0.5 ml of CRE-Kpn19 (4.1 x 10^7^ UFC/mL) and treatments started 4 h post-infection (hpi), according to their respective group: (i) control treated with placebo; (ii) a single IV dose of fluopsin C at 1 mg/Kg; (iii) a single IV dose of fluopsin C at 2 mg/Kg; (iv) a single IV dose of fluopsin C at 3 mg/Kg; (v) two IV doses of fluopsin C at 1 mg/Kg, 8 h apart; (vi) two IV doses of fluopsin C at 2 mg/Kg, 8 h apart. Mortality was recorded over 96 hpi.  For the histopathological analysis, cohorts of 2 mice were euthanized at 1, 10, 20, 40 days after treatment with one IV dose of fluopsin C at 2 mg/Kg or placebo. Kidney and liver were excised and fixed in 10% neutral buffered formaldehyde. | Experimental Murine Model |
| **Experimental animals** | 8 | The mice used was immunocompetent female Swiss albine (7 ± 1 week old and 32 ± 3 g) obtained of the central animal facility of the UEL and acclimatized in the laboratory for at least 48 h. | Experimental Murine Model. |
| **Housing and husbandry** | 9 | Animals were maintained in polypropylene boxes with wood shaving bedding and provided with sterilized water and commercial feed (Nutival®) throughout the experiment*.* The mice were kept in ventilated shelving with temperature (24 °C), humidity of (55%) and photoperiod of 12/12 hour controlled. | Experimental Murine model: |
| **Sample size** | 10 | Sixty-four healthy mice divided into three experiments: eighteen for dose determination and lethal inoculum; thirty-six animals to determine efficacy; and ten animals for histopathology studies. The experiment was repeated. | Experimental Murine Model |
| **Allocating animals to experimental groups** | 11 | For experiments using animal, search box with mice group were allocated to treatment, i.e. Control group, Treatment 1 (4 mg/Kg), Treatment 2 (2 mg/Kg), etc., | Experimental Murine Model |
| **Experimental outcomes** | 12 | Survival was the primary outcome measured. In addition, histological material was collected for analysis. | Experimental Murine Model |
| **Statistical methods** | 13 | Statistical analyses and graphics were performed by using R software (R Core Team, 2014). Arranged in a survival curve, using the Kaplan–Meier estimator. | Statistical analysis |
| **Results** |  |  |  |
| **Baseline data** | 14 | The animals’ health status was monitored throughout the experiments by observation in periods of 4 to 12 h.   | Experiment | Health status | Monitored Time | Periods | | --- | --- | --- | --- | | Lethal Doses | Drug inoculation | 96h | 4 to 12 h | | Lethal Inoculum | Infection with *K. pneumoniae* | 96 h | 4 to 12 h. | | Efficacy | Infection with *K. pneumoniae* and Drug inoculation | 96 h | 8 to 24 h. | | Histopathology | Drug inoculation | 40 days | 1 - 10 days | | Antibiotic Activity Evaluation in Mice Model |
| **Numbers analysed** | 15 | The lethal dose (LD) of fluopsin C was determined, where concentrations below 4 mg/Kg (LD_50_, 3/6) was not toxic for mice after 96h (0/6), while higher doses produced lethality starting after 48h (6/6).  The animals than received an IP inoculum of 0.5 ml of CRE-Kpn19 (4.1 x 10^7^ UFC/mL). Control, animals not treated with fluopsin C, died before of 96h (11/12).  Mice treated with a single dose of 2 mg/Kg and two doses of 1 mg/Kg/8h (treatments iii and v, respectively) showed a survival of 50% (6/12) and 42% (5/12) after 72h of treat with fluopsin C respectively. The group treated with one dose of 1 mg/Kg (treatment ii), showed a survival rate of 25% (3/12) after 48h and 17% (2/12) after 96h. Groups iv and vi (3 mg/Kg and two doses of 2 mg/kg/8h, respectively) presented low survival rates, reaching complete mortality (12/12) even faster than control group. | Toxicity of Fluopsin C in Alternative Model / Figure 5 |
| **Outcomes and estimation** | 16 | Not applicable |  |
| **Adverse events** | 17 | No |  |
| **Discussion** |  |  |  |
| **Interpretation/scientific**  **Implications** | 18 | Despite of the negligible bioavailability presented by fluopsin C, it increased the survival rate of Swiss mice infected with *kpn-*KPC 19, thereby protecting a high percentage of the infected animals against sepsis. This is the first report of fluopsin C efficacy on MDR infected mice. In addition, fluopsin C did not cause pathological alterations in kidney cells, such as glomerular corpuscles and the Bowman´s space area, suggesting that fluopsin C does not have nephrotoxicity. On the other hand, it increased hepatocytes with vacuolated cytoplasm and displaced nucleus. | Discussion |
| **Generalisability/ translation** | 19 | The strong antibiotic activity of fluopsin C under *in vitro* and *in vivo* conditions stressed the power of this compound to become an alternative against Gram-positive and Gram-negative MDR infections, despite the cytotoxicity observed. Fluopsin C have a great potential to be explored and may soon be used in further preclinical assays. | Discussion |
| **Funding** | 20 | This work was supported by The Brazilian National Council supported this work for Scientific and Technological Development and Coordination for the Improvement of Higher Level -or Education- Personnel, Brazil. | Funding |
